# Trust Barriers and Vulnerabilities in Older Adults’ Telemedicine Adoption: Scoping Review

**DOI:** 10.2196/84818

**Published:** 2026-07-14

**Authors:** Wanqing Wang, Xin Ma, Matthew Ting Chi Liu, Angela Chang

**Affiliations:** 1 Department of Communication, Faculty of Social Sciences, University of Macau, Avenida da Universidade, Taipa, Macau, China Special Administrative Region, 853 88228991; 2 Department of Management and Marketing, Faculty of Business Administration, University of Macau, Macau, China

**Keywords:** trust, vulnerability, telemedicine, older adults, digital health, health equity

## Abstract

**Background:**

Aging populations worldwide face increasing health care demands, particularly for chronic disease management. While telemedicine offers a viable solution to enhance health care access, significant trust-related barriers hinder its adoption among older adults, warranting a scoping synthesis of available evidence.

**Objective:**

This scoping review aims to map the available evidence on trust barriers experienced by older adults in telemedicine, identify underlying vulnerability domains, and chart the evidence base for design and policy recommendations.

**Methods:**

We conducted a scoping review in accordance with PRISMA-ScR (Preferred Reporting Items for Systematic Reviews and Meta-Analyses Extension for Scoping Reviews) guidelines, analyzing literature from PubMed, Web of Science, and Scopus. Thematic analysis was applied to synthesize findings from 30 included studies.

**Results:**

Four primary trust barriers were identified: technophobia and technical difficulties, privacy and data security concerns, negative emotional and social impacts, and a strong preference for in-person care. These barriers mapped onto 4 vulnerability domains: limited telemedicine literacy (particularly low eHealth self-efficacy), declining health status (including sensory and cognitive impairments), psychological and cognitive factors (such as anxiety about losing autonomy), and inadequate social support systems. The review also underscored how rapid technological change amplifies these challenges for older adults.

**Conclusions:**

Effective telemedicine implementation for older adults requires multipronged interventions, including age-appropriate interface design, targeted digital literacy training, robust privacy protections, and personalized support systems. These approaches address both technological and psychosocial barriers, potentially increasing engagement while mitigating vulnerabilities. Future research should assess the effectiveness of these interventions across diverse older populations.

## Introduction

### Background

As demographic transitions advance, characterized by declining fertility and increasing mortality in most countries, along with increased life expectancy due to the decline in deadly infectious diseases, there has been a remarkable increase in global population aging. It is estimated that by 2050, the worldwide aging rate will rise to 15.9%, with projections indicating it could reach 22.4% by the year 2100 [1,2]. Aging populations present a growing health care challenge, as older adults often require care for chronic diseases such as heart conditions, diabetes, or respiratory issues. Traditional in-person health care, hindered by mobility limitations, transportation difficulties, and frequent appointments, is often inaccessible, while telemedicine offers a promising alternative to enhance convenience [3-5].

Telemedicine can be broadly defined as the use of telecommunication technologies to provide medical information and services [6-9]. In practice, it encompasses a suite of resources designed to facilitate communication between patients and health care providers, including online consultations, remote monitoring, and virtual rehabilitation. Nevertheless, addressing trust-related challenges is essential for the widespread adoption of telemedicine, particularly among older adult populations [7,10-12].

Trust is commonly defined as the willingness of trustors to accept vulnerability, and the level of vulnerability at the individual level is key to understanding trust [13-15]. Perceived trust issues have been documented in existing research among older adults, including the use of digital health technologies [9,16,17], concerns about information privacy [18,19], and doctor-patient interaction [14-16]. Trust deficits among older adults have been identified as substantive obstacles to telemedicine uptake, limiting service acceptance and access to health care [20,21].

While prior studies have highlighted the trust issues that older adults encounter in telemedicine, a critical knowledge gap remains. Although trust is commonly defined as an acceptance of vulnerability [13-15], more research has emphasized surface-level factors underlying trust concerns. The deeper dimensions of vulnerability that directly shape trust perceptions remain underexplored. Because vulnerability is not simply an outcome but an intrinsic element of trust dynamics, it cannot be overlooked. In this review, we conceptually distinguish trust barriers as the observable manifestations of distrust or resistance in the context of telemedicine, and vulnerabilities as the underlying predisposing states or conditions that create susceptibility to these barriers. This distinction follows Hamm et al [13], who define vulnerability as an intrinsic antecedent of trust dynamics rather than an outcome. Without clarifying how specific vulnerabilities manifest and interact among older adults, strategies to enhance trust in telemedicine risk lacking the precision necessary to dismantle entrenched barriers and improve health care access for this population.

### Objective

This scoping review aims to map the available evidence on trust barriers in older adults’ telemedicine use; chart how these barriers link to underlying vulnerability dimensions, and as an exploratory extension; and identify practice-informed design recommendations. At present, in the field of gerontechnology and digital health, it remains unclear how trust-eroding factors in telemedicine are interrelated with the vulnerabilities of older adults at the individual, interpersonal, and system levels.

To achieve this, the study focuses on 4 interconnected research questions (RQs), informed by established digital health frameworks (eg, the Technology Acceptance Model for Mobile Health) [22,23] and extended to center on dimensions of vulnerability:

- RQ1: What evidence exists regarding the adoption of telemedicine among older adults in existing research?
- RQ2: What trust-eroding factors in telemedicine are documented among older adults, and how do they vary across individual, interpersonal, and systemic levels?
- RQ3: How do these trust-eroding factors correlate with specific dimensions of vulnerability at the individual, interpersonal, and systematic levels?
- RQ4: What intervention strategies can address these vulnerabilities and enhance trust in telemedicine?

These questions operationalize a multilevel analytical lens, encompassing the individual level (eg, declining health status, sensory impairments, and limited digital literacy) [24,25]; the interpersonal level (eg, patient-provider communication patterns [26,27], caregiver support, and peer influences on technology perceptions); the systemic level (eg, data privacy regulations, platform design standards, and the availability of technical support infrastructure) [24,25,28-30]; and the cumulative interactions across levels [31,32].

By synthesizing the current state of knowledge, this study aims to advance theoretical understanding of how vulnerability shapes trust dynamics in aging populations. It moves beyond surface-level trust factors to examine their root causes within layered vulnerabilities. Practically, it seeks to inform scalable, evidence-based interventions that enhance telemedicine acceptance and effectiveness, bridging empirical insights with real-world solutions.

## Methods

### Overview

A scoping review, combined with thematic analysis, was conducted to examine trust barriers experienced by older adults in telemedicine and their associated vulnerabilities. Scoping reviews are valuable for rapidly mapping key concepts, primary sources, and evidence types within a research area, particularly in complex or underexplored fields [33,34]. This approach is especially effective for (1) identifying the scope of available literature on a specific topic, (2) providing an overview of key concepts, and (3) identifying gaps in the research landscape [35-39]. In contrast, systematic reviews primarily synthesize quantitative outcomes to assess intervention effectiveness [38-40].

Given the fragmented and limited research on the determinants of trust and associated vulnerabilities in telemedicine adoption among older adults, a scoping review with thematic analysis was selected as the methodological approach. Existing studies have largely emphasized surface-level trust issues without systematically exploring the underlying dimensions of vulnerability that shape these perceptions. This omission has left a significant gap in understanding how technological, informational, and relational vulnerabilities interact to influence trust in telemedicine. To address this gap, the combined scoping review and thematic analysis was deemed most appropriate.

Thematic analysis was employed to analyze the qualitative data extracted from the included studies. This approach offers a flexible yet rigorous method for analyzing textual data, enabling the identification, analysis, and reporting of patterns (themes) within the data [40,41]. Thematic analysis was chosen to provide a rich, nuanced understanding of the factors influencing older adults’ trust in telemedicine. Examples of studies employing a combined scoping review and thematic analysis approach exist in diverse fields such as medicine, education, and sociology [42-45]. Thematic analysis followed an inductive approach [46], proceeding in 6 stages: familiarization with data, generating initial codes, searching for themes, reviewing themes, defining and naming themes, and producing the report. Initial coding was performed by the lead author. To establish interrater reliability, the second author independently coded approximately 30% of the extracted excerpts. Interrater agreement was substantial (Cohen κ=0.78) [47]. Discrepancies were resolved through consensus discussion with all of the coauthors. Thematic saturation was considered achieved when the final 5 studies reviewed yielded no new codes.

The study was conducted and reported in accordance with the PRISMA-ScR (Preferred Reporting Items for Systematic Reviews and Meta-Analyses Extension for Scoping Reviews) guidelines (Checklist 1), an evidence-based framework consisting of a 4-phase flow diagram and a 27-item checklist to ensure methodological rigor and transparency in scoping reviews [48-50].

### Searching and Screening Process

Electronic searches for this scoping review were conducted in May 2025, timed with the World Health Organization (WHO) Global Strategy on Digital Health 2020 to 2025, post its mid-2025 progress review to capture up-to-date evidence on older adults’ digital health. Three significant databases were considered as primary sources, namely PubMed, Web of Science, and Scopus, as they include subjects such as health, aging, medicine, digital technology, communication, and psychology.

Before finalizing the search strategy, we explored the 3 databases using key concepts such as “telehealth,” “trust,” and “vulnerability.” Keywords were expanded based on the initial search results. Next, a unified search term incorporating Boolean operators was utilized across all databases:

((((trust AND (vulnerability OR vulnerable)) AND (telemedicine OR telehealth OR eHealth OR mHealth)) AND (elderly OR older adults OR old people)) AND patient).

In PubMed, searches were conducted using both free-text keywords and MeSH terms (eg, “Telemedicine” [MeSH], “Aged” [MeSH], and “Trust” [MeSH]) to ensure comprehensive coverage. The search covered all publication years and was limited to English-language sources. To ensure methodological rigor, only peer-reviewed journal papers and review papers were included.

Inclusion criteria were as follows: (1) studies must directly address trust issues or the vulnerability of old patients in telemedicine; (2) study participants must be aged 55 years or older, or include a dedicated analysis of a group over 55 years old. Reviews with unreported demographics were included only if 75% or more of their synthesized studies explicitly focused on older adults (eg, through keywords such as “geriatric” or “aging”); and (3) studies must be patient-centered and conducted from the patients’ perspective.

Exclusion criteria included (1) non–peer-reviewed and nonscholarly materials, such as conference papers, book chapters, reports, design work, personal interview reports, newspaper articles, and other nonacademic publications; (2) studies with missing data or requiring paid access beyond institutional subscriptions, due to practical accessibility limitations; and (3) on PubMed, papers not based on human subjects were specifically excluded. These criteria ensure that the review’s findings directly inform its research questions regarding enhancing trust in telemedicine among older adults.

Overall, the search yielded 740 publications across 3 databases, which was reduced to 723 after duplicates were removed using EndNote 21.4 (Clarivate Analytics). The first author independently screened titles and abstracts for relevance, followed by a second round of eligibility assessment. Of these, 253 studies met the inclusion criteria and proceeded to full-text review.

Papers that did not focus on older patients aged 55 years or older or did not discuss issues from the patients’ perspective were excluded (86/723, 11.9%), as were papers that did not capture trust issues or the vulnerability of older patients in telemedicine (57/723, 7.9%). In addition, we discarded papers that only briefly mentioned trust and vulnerability without exploring these concepts in depth (80/723, 11.1%). Specifically, studies that referenced trust and vulnerability in passing—such as in the introduction or discussion—without providing a focused analysis, dedicated sections, or empirical evidence directly addressing these concepts were excluded. This decision was made by assessing the relevance of trust and vulnerability to the study objectives, methodologies, and findings.

Data extraction was performed using a standardized charting form developed iteratively. The form was piloted on 5 randomly selected included studies and refined by the research team to ensure completeness and consistency. The final charting form captured the following variables for each included study: (1) author(s) and year of publication, (2) journal and study design, (3) participant age range and sample size, (4) geographic focus and country, (5) telemedicine modality, (6) specific trust barriers identified, and (7) vulnerability dimensions reported. Two independent reviewers performed data extraction, and discrepancies were resolved through discussion or consultation with a third reviewer. Extracted data were cross-verified by both reviewers.

Findings were synthesized using inductive thematic analysis, following the six-phase framework described by Braun and Clarke [46]: (1) familiarization with the data through repeated reading of the extracted text, (2) generating initial codes applied to trust-barrier and vulnerability-related content, (3) searching for themes by grouping related codes, (4) reviewing themes for coherence and distinctiveness, (5) defining and naming final themes, and (6) producing the synthesis report. Codes and emerging themes were discussed among coauthors to enhance interpretive rigor.

## Results

### Characteristics of Included Studies

**Figure 1.**
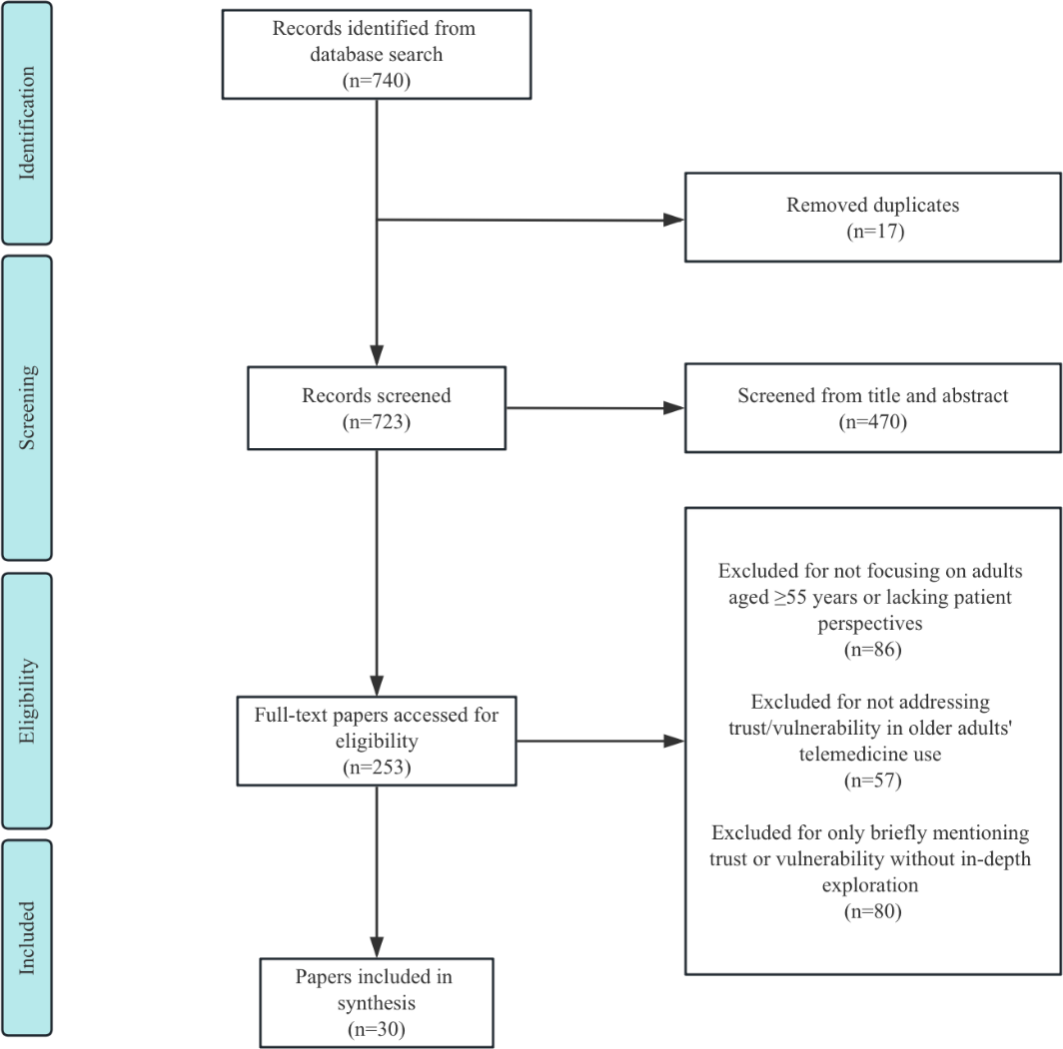
PRISMA-ScR (Preferred Reporting Items for Systematic Reviews and Meta-Analyses Extension for Scoping Reviews) flow diagram of the study selection process for the scoping review.

The final sample comprised 30 studies, of which 28 (93.3%) were published between 2020 and 2025 and only 2 (6.7%) prior to 2020. This dominance of post-2020 publications reflects the rapid expansion of telemedicine research during the COVID-19 pandemic, as the public health crisis heightened the need for remote health care solutions. The included studies consisted of quantitative research (11/30, 36.7%), qualitative research (8/30, 26.7%), mixed-methods research (1/30, 3.3%), and reviews (10/30, 33.3%). The 11 quantitative studies (typically large cross-sectional surveys using validated scales) provide the strongest evidence for the relative prevalence of specific barriers. The 8 qualitative studies offer contextually rich descriptions of lived trust experiences, capturing mechanisms that quantitative measures may not fully detect. The 10 review papers (comprising 5 scoping reviews, 2 systematic reviews, 1 rapid review, 1 realist review, and 1 narrative review) synthesize broader evidence but introduce additional heterogeneity across populations and contexts. The descriptive characteristics of all included studies are presented in Multimedia Appendix 1. A total of 30 papers were included in the review, and the extraction procedures are presented in Figure 1.

### Geographic and Economic Distribution of Studies

The included studies were conducted across 12 countries, with China contributing the largest number (7/30, 23.3%), followed by the United Kingdom (3/30, 10%) and the United States (2/30, 6.7%). Other contributing countries included Germany, Sweden, the Philippines, Malaysia, India, Australia, New Zealand, and Finland (each with 1/30, 3.3%). Geographic coverage revealed a stark imbalance: most studies (28/30, 93.3%) focused on high-income regions (Europe, North America, and parts of Asia), with very few addressing low- and middle-income countries, underscoring a global equity gap.

Population size varied by study design. Quantitative studies had larger samples (111‐1852 participants), whereas qualitative studies (including mixed-methods research) had smaller samples (6‐98 participants). For review papers, the number of included studies ranged from 10 to 193, except for 1 narrative review that did not report a specific sample size.

Although the general threshold for older adults is often 65 years or more (WHO standard: ≥60), more than a quarter of the included studies (8/30, 26.7%) expanded this range. Specifically, 18 (60.0%) papers reported an age range of 60 years old and above, whereas 8 (26.7%) papers broadened the range to 55 years old and above; 2 (6.7%) papers did not report precise age ranges (one reported an average age of 66.94 years, and the other reported the majority were over 65 years). Another 2 (6.7%) reviews did not report the age range but were also retained after verifying that over 75% (22/30) of their original studies focused on the older adult population. Researchers cited several reasons for the expansion: (1) 50 or more is a common threshold in technology-use literature, reflecting demographic trends; (2) widening the range avoided excluding high-quality studies and supported inclusive evidence; (3) in some populations (eg, the homeless), 50 or more is recognized as older due to “accelerated aging;” and (4) alignment with the global minimum retirement age of 50 years, which accounts for socioeconomic dimensions of aging.

Across the 30 included studies, telemedicine modalities varied, with 5 common types identified: telemedicine apps (11 papers), wearable devices (10 papers), video consultations (5 papers), electronic surveillance (4 papers), and government services (4 papers). Other modalities included social media services, family therapy, virtual diagnostics, monitoring sensors, SMS text messaging, and online counseling (each reported in 1‐2 papers). Six papers did not specify the modality. Table 1 provides the detailed evidence on older adults’ telemedicine adoption.

**Table 1.** Characteristics of older adults’ telemedicine adoption^a^.

Number	Authors and year	Methods (study design)	Participants’ age (y)	Sample size		Geographic focus (country)	Telemedicine modality
				Empirical research sample	Review included studies		
1	Essén [51] (2008)	Qualitative research	≥68	17	—^b^	Sweden	Electronic surveillance
2	Fitzsimmons et al [52] (2016)	Qualitative research	Mean=66.94	9	—	The United Kingdom	Wearable devices
3	Kruse et al [53] (2020)	Systematic review	≥50	—	57	Europe and America	Video consultations, electronic surveillance, and telemedicine apps
4	Ladin et al [54] (2021)	Qualitative research	≥70	30	—	The United States	No specified
5	Schorr et al [55] (2021)	Systemic review	≥65	—	26	Europe, North America, and Asia	Telemedicine apps, wearable devices, and SMS text messaging
6	Wilson et al [25] (2021)	Scoping review	≥60	—	14	Europe, America, and Australia	Telemedicine apps and online counseling
7	Chen and Liu [56] (2022)	Quantitative research	≥50	390	—	China	Wearable devices
8	Hunter et al [57] (2022)	Qualitative research	≥55	98	—	New Zealand	No specified
9	Kaihlanen et al [21] (2022)	Qualitative research	≥65	16	—	Finland	Government service
10	Singh et al [58] (2022)	Quantitative research	≥60	534	—	India	Wearable devices
11	Korkmaz Yaylagul et al [59] (2022)	Scoping review	≥65	—	40	Europe, North America, and Asia	Video consultations, electronic surveillance, telemedicine apps, and wearable devices
12	Guo et al [17] (2023)	Quantitative research	≥50	111	—	China	Telemedicine apps
13	Liu et al [11] (2023)	Quantitative research	≥65	201	—	China	Telemedicine apps
14	Orzechowski et al [60] (2023)	Qualitative research	≥73	11	—	Germany	Wearable devices
15	Ali et al [61] (2024)	Qualitative research	≥65	6	—	The United Kingdom	Telemedicine apps
16	Chen et al [9] (2024)	Quantitative research	≥60	309	—	China	No specified
17	Chu et al [62] (2024)	Quantitative and qualitative research	Survey: 68 (SD 11); Interview: 65 (SD 16)	55	—	Australia	Family therapy
18	Fang et al [63] (2024)	Quantitative research	≥60	277	—	China	Virtual diagnostics, telemedicine apps, and wearable devices
19	Gallardo et al [7] (2024)	Quantitative research	≥60	180	—	The Philippines	Telemedicine apps
20	Li et al [2] (2024)	Quantitative research	≥60	400	—	China	No specified
21	Niu et al [64] (2024)	Quantitative research	≥60	661	—	China	Government service
22	Turcotte et al [65] (2024)	Rapid review	≥50	—	24	Europe, North America, and Asia	Telemedicine apps, video consultations, and wearable devices
23	Windle et al [66] (2024)	Scoping review	Most≥65	—	193	Europe, North America, and Australia	No specified
24	Husain and Greenhalgh [67] (2025)	Qualitative research	≥65	17	—	The United Kingdom	Video consultations
25	Tan et al [68] (2025)	Quantitative research	≥60	119	—	Malaysia	Video consultations, wearable devices, and government service
26	Zhu et al [69] (2025)	Quantitative research	≥60	1852	—	The United States	No specified
27	Adams et al [70] (2025)	Scoping review	≥50	10	—	America	Telemedicine apps and government service
28	Fothergill et al [30] (2025)	Realist review	Unreported	—	10	Europe, America, and Australia	Pendant alarms, monitoring sensors, wearable devices, and electronic surveillance
29	Western et al [29] (2025)	Narrative review	Unreported	—	Unreported	Global	Social media service, telemedicine apps, and wearable devices
30	Zhang et al [71] (2025)	Systematic review	≥50	—	16	Asia and North America	Social media service and telemedicine apps

a Two reviews lacked explicit reporting but were retained after verifying that 75% or more of their primary studies targeted older populations.

b Not applicable.

### Dimensions of Trust Barriers

Analysis of the included studies identified 4 thematic categories of trust barriers: technophobia and difficulties, preference for in-person care, privacy and data concerns, and emotional and social impacts.

#### Technophobia and Difficulties

Technical issues were the most prevalent barrier, reported in 28 (93.3%) papers. Subthemes included low technical skills (eg, inability to navigate interfaces and limited understanding of monitoring mechanisms; 19/30, 63.3%), lack of technical support (eg, absence of family assistance and inadequate troubleshooting services; 14/30, 46.7%), age-related sensory or cognitive decline (eg, impaired vision or hearing and reduced attention span; 12/30, 40.0%), and technical anxiety or fear of using electronic devices (9/30, 30.0%). Less common but notable factors included physical coordination limitations (eg, motor impairments affecting device use; 4/30, 13.3%) and inconsistent user interfaces across platforms (2/30, 6.7%). Modality-specific patterns were evident: studies involving telemedicine applications (11/30, 36.7%) most frequently identified barriers related to interface complexity and low self-efficacy, with participants reporting difficulty navigating functions, performing self-monitoring tasks, or evaluating information reliability. Studies involving wearable devices (10/30, 33.3%) emphasized physical operation difficulties and doubts about data accuracy, reflecting the additional dexterity and technical comprehension demands of sensor-based technologies.

#### Preference for In-Person Care

A strong preference for in-person care was another significant barrier, identified in 26 (86.7%) papers. Specifically, 18 (60.0%) papers directly reported a preference for in-person care; 15 (50.0%) noted concerns about the accuracy of telehealth diagnostics or equipment; and 11 (36.7%) indicated reliance on manual verification processes. Low health literacy, contributing to skepticism toward telemedicine, was reported in 5 (16.7%) papers. Cultural influences were highlighted in 3 papers, pointing to disparities in the acceptance of innovative medical approaches across Eastern and Western contexts or between ethnic groups. Studies (4/30, 13.3%) examining government digital health services additionally identified complex digital pathways, inadequate multilingual support, and structural access inequities as barriers reinforcing this preference, suggesting that system-level design failures can compound individual reluctance toward remote care.

#### Privacy and Data Concern

Privacy and data issues were identified in 23 (76.7%) papers. Concerns included fear of data leakage or breaches (16/30, 53.3%), perceived intrusiveness of continuous monitoring (10/30, 33.3%), and lack of ownership or control over data, including uncertainty about sharing practices (5/30, 16.7%). In a few cases, lack of personal devices or internet access contributed to privacy-related distrust (3/30, 10.0%). Studies involving electronic surveillance technologies (4/30, 13.3%) generated the most acute privacy concerns, encompassing fears about unauthorized third-party access, stigma associated with being continuously monitored, and the perceived intrusiveness of ambient sensors tracking behavioral patterns. Studies involving wearable devices similarly surfaced a concern specific to passive, always-on data collection. However, an exception was noted in a Swedish study on electronic care surveillance, where most participants (16/17) viewed the service positively, citing safety benefits, while only 1 participant expressed discomfort with constant monitoring.

#### Emotional and Social Impacts

Emotional and social impacts were identified in 21 (70%) papers. Reported issues included discomfort in virtual settings, such as receiving distressing news without perceiving empathy or lacking nonverbal cues (6/30, 20.0%). Concerns about losing personal connections with clinicians were expressed in 12 (40.0%) papers, and refusal to engage due to stigma or embarrassment—such as perceiving telemedicine use as a sign of dependence or incompetence—was noted in 7 (23.3%) papers. Specifically, in 1 (3.3%) mental health study, participants expressed uncertainty about the identity of the remote provider, which heightened distrust. A few secondary factors (eg, financial costs for users and organizational resource constraints in rural areas) were also noted but lacked sufficient empirical support for generalizable conclusions. This barrier was most documented in studies focusing on video consultations (5/30, 16.7%). In synchronous video communication, participants described an intensified sense of detachment resulting from screen-mediated interaction and a reduction in nonverbal cues. In contrast, emotional concerns arising from asynchronous communication primarily centered on a lack of human warmth and continuity.

### Dimensions of Vulnerabilities

Across the 30 included studies, 4 primary categories of vulnerabilities related to telemedicine adoption among older adults were identified: low telemedicine literacy, dependence on social support, psychological and cognitive dispositions, and complex health conditions.

#### Low Telemedicine Literacy

Low telemedicine literacy was the most frequently documented vulnerability, identified in 29 (96.7%) papers. Three dominant subthemes emerged within this category. Limited knowledge of telehealth devices and services was reported in 22 (73.3%) studies, including insufficient understanding of device functions, data collection mechanisms, and service scope. Dependence on external guidance was observed in 16 (53.3%) studies, with participants requiring assistance from family members, caregivers, or clinical staff to operate devices, navigate platforms, or interpret data. Low self-management skills were noted in 14 (46.7%) studies, reflected in difficulties independently monitoring personal health indicators (eg, blood glucose and blood pressure) or adjusting health behaviors based on remote feedback.

Exceptional cases included a lack of access to telemedicine training resources (eg, absence of community workshops and insufficient user manuals), as reported in 4 (13.3%) studies. Additionally, 3 (10%) papers highlighted language barriers, such as limited English proficiency or the inability to describe symptoms without relying on nonverbal communication.

#### Dependence on Social Support

Dependence on social support was as prevalent as low telemedicine literacy, identified in 29 (96.7%) studies. This dependency manifests in 3 aspects. Reliance on family members or caregivers for device operation was reported in 21 (70.0%) studies. Participants required assistance with device setup (eg, pairing wearables with smartphones), troubleshooting technical issues, or entering health data. Dependence on clinical staff for health management was noted in 17 (56.7%) studies, encompassing reliance on clinicians for medication adjustments, interpretation of telehealth data, and guidance on disease management through remote channels. Living alone with limited immediate support was observed in 14 (46.7%) studies, where solitary living arrangements heightened concerns about delayed assistance during acute health events (eg, falls and sudden symptom exacerbations) when using telemedicine. Three (10.0%) papers suggested that older adults may lack intrinsic health behavioral drivers and rely more on external incentives to motivate participation in health-related activities.

Apart from artificial assistance, 5 (16.7%) papers reported financial dependency as a barrier to telemedicine access, such as the inability to afford devices or internet services. Additionally, 3 papers indicated difficulties accessing general practitioners due to structural barriers (eg, appointment shortages and geographic inaccessibility).

#### Psychological and Cognitive Disposition

Alongside factors of efficiency and convenience, the reviewed literature emphasized the importance of mental elements in building trust. Vulnerabilities related to psychological and cognitive disposition were noted in 22 (73.3%) studies. The most common subtheme was anxiety or depression related to disease uncertainty (15/30, 50%), with participants experiencing emotional distress over disease progression or the reliability of remote diagnosis. Cognitive impairment affecting decision-making was reported in 10 (33.3%) papers, encompassing memory decline, reduced attention span, and difficulty comprehending medical information—factors that hindered engagement with telemedicine processes (eg, scheduling appointments, following remote care plans). Low self-esteem or grief-related distress appeared in 6 (20%) studies, where negative self-perceptions (eg, feeling “technologically incompetent”) or grief (eg, loss of independence) further reduced willingness to adopt telemedicine.

#### Complex Health Conditions

Older adults’ prevalent physical disabilities and complex health conditions present distinct vulnerabilities within the telemedicine context (19/30, 63.3%), manifesting through 3 interconnected dimensions.

Twelve (40%) papers addressed older adults’ chronic multimorbidity, including concurrent conditions such as diabetes, hypertension, and cardiovascular disease, which increased the complexity of remote health monitoring and management. Ten (33.3%) papers reported progressive physical decline, characterized by age-related frailty, reduced mobility, and heightened fall risk—factors that limited independent use of telemedicine devices (eg, wearable sensors and video consultation tools). Seven (23.3%) papers included complications requiring frequent care adjustments, such as acute exacerbations of chronic obstructive pulmonary disease or kidney disease, which necessitated timely in-person clinical intervention and reduced reliance on telemedicine alone. Older patients’ heightened awareness of health risks and self-perceived vulnerability drive stringent expectations for health care accuracy, timeliness, and reliability (2/30, 6.7%).

Table 2 presents the co-documentation frequencies between the 4 trust barrier themes and 4 vulnerability dimensions across the 30 included studies. Each cell indicates the number of studies that simultaneously reported both constructs, based on the thematic coding documented in Multimedia Appendix 1. The high co-documentation frequencies across most cells reflect the interconnected nature of these constructs: vulnerabilities rarely map onto a single barrier in isolation, and multiple vulnerability dimensions frequently co-occur with each trust barrier theme within the same study. These patterns are consistent with the narrative findings, which emphasize that the relationships between trust barriers and vulnerabilities are layered and nonlinear rather than reducible to discrete one-to-one pairings.

**Table 2.** Co-documentation frequency matrix: trust barrier themes×vulnerability dimensions across included studies (N=30)^a^.

Dimension	T1: technophobia and technical difficulties (n=28), n (%)	T2: preference for in-person care (n=26), n (%)	T3: privacy and data concerns (n=23), n (%)	T4: emotional and social impacts (n=21), n (%)
V1: low telemedicine literacy (n=29)	27 (90.0)	25 (83.3)	22 (73.3)	21 (70.0)
V2: dependence on social support (n=29)	27 (90.0)	25 (83.3)	22 (73.3)	20 (66.7)
V3: psychological and cognitive disposition (n=22)	22 (73.3)	19 (63.3)	18 (60.0)	16 (53.3)
V4: complex health conditions (n=19)	18 (60.0)	16 (53.3)	14 (46.7)	13 (43.3)

a Cell values represent the number (and percentage) of included studies simultaneously documenting each trust barrier-vulnerability pair, based on thematic codes in Multimedia Appendix 1. Marginal totals in parentheses indicate how many studies documented each construct independently; co-documentation frequencies should be interpreted in relation to these base rates. No directionality or causal relationship is implied.

## Discussion

### Principal Findings

The most significant finding of this scoping review is the identification of 4 interrelated vulnerability dimensions—technological literacy, health status, psychological or cognitive dispositions, and social support—that collectively underpin trust erosion in older adults’ telemedicine use. By synthesizing evidence from previously fragmented studies, this review demonstrates that these vulnerabilities consistently align with 4 clusters of trust barriers: technophobia, privacy concerns, emotional and social impacts, and preference for in-person care. These vulnerabilities operate across 3 interconnected levels: at the individual level, low technological literacy and adverse psychological dispositions restrict autonomy; at the interpersonal level, diminished social support and technology dependence shift erode dignity and emotional validation; and at the systemic level, gaps in platform accessibility, privacy safeguards, and integration with in-person care exacerbate digital exclusion.

Together, these findings offer an integrative and organizing framework that consolidates previously fragmented evidence into a multilevel map of how vulnerability shapes trust dynamics in older adults’ telemedicine use. Additionally, the synthesis provides a practical scaffold for targeted intervention design. Corresponding strategies were identified at each level, as detailed in subsequent sections.

### Implications of Telemedicine Trust

Building on this framework, the evidence suggests that older adults’ trust in telemedicine is eroded through layered “loss of control”—rooted in interconnected vulnerabilities across all 3 levels [64,72,73].

At the individual level, the loss of bodily control is central. Declining physical functioning increases reliance on technological devices and caregiver assistance, eroding bodily and informational autonomy. Telemedicine tools often feel intrusive (eg, forced disclosure of sensitive information) or impersonal, with standardized services failing to address complex needs. Uncertainty about remote diagnosis further weakens perceived control over health outcomes, directly undermining trust [73,74].

At the interpersonal level, the loss of identity control is pronounced. As older adults withdraw from the labor market, dependence on others for technology use redefines them from “independent” to “cared-for,” threatening dignity—particularly among marginalized groups (eg, low educational attainment) [75-77]. Unlike in-person care, virtual interactions reduce emotional validation and social role confirmation, leading to feelings of loneliness, neglect, or diminished attachment [78,79].

At the systemic level, the loss of generational control reflects broader technological shifts. Telemedicine amplifies “digital exclusion” for older adults who are unfamiliar with platforms or cybersecurity, triggering anxiety [53,80,81]. Generational gaps in technological exposure deepen this divide: many individuals prefer in-person care, feeling sidelined in a system that prioritizes digital innovation over familiar health care relationships, further exacerbating their vulnerability [63,82].

In summary, the factors tied to individual autonomy, interpersonal identity, and systemic adaptability demonstrate that trust cannot be addressed through isolated fixes. The multilevel framework provides a cohesive foundation for targeted interventions across all 3 levels. Since this study broadens the age-based definition of “older adults,” this framework is better suited as a descriptive map applicable to the broader older adult spectrum rather than a precision model for any single age cohort. In practice, clinicians and policymakers should develop tailored strategies for specific age subgroups rather than treating “older adults” as a homogeneous category.

### Enhancing Inclusive Telemedicine

Translating the multilevel framework into practice, the following recommendations address trust barriers at each level, organized by the strength of evidence.

First, design and training can directly reduce individual barriers to trust. The included studies identified interface complexity and insufficient training as primary drivers of technophobia. To address technological vulnerabilities, telemedicine platforms should prioritize age-friendly interfaces with large fonts, voice-activated commands, and modular operation guides to improve usability [83-85]. Crucially, the included studies also emphasized that on-demand technical support must accompany these tools, as older adults’ reluctance to engage often stems not from fixed incapacity but from a fear of making errors without accessible help.

Second, humanizing interactions can strengthen interpersonal connections, which are essential for trust. Multiple studies have identified the loss of the therapeutic relationship and the absence of nonverbal communication as central to emotional distrust in video consultations, pointing to the need for provider training in virtual communication skills. Training providers to use nonverbal cues (eg, nodding and expressive facial gestures) during video consultations adds warmth to virtual visits [86,87]. The evidence also consistently highlights the role of family members and informal caregivers. Involving authorized family members in virtual visits and providing shared record access can reduce recovery anxiety and sustain engagement, thereby reinforcing trust [88,89].

Third, systemic improvements must anchor these efforts, encompassing privacy, cultural adaptability, and accessibility [90,91]. Studies across multiple contexts identified privacy transparency as a foundational trust mechanism. To protect privacy, telemedicine platforms should provide visual guides that clarify the scope of data collection and enable users to customize sharing permissions through intuitive privacy dashboards [91-93]. Sensitive data (eg, mental health records or sexual health information) require double encryption to establish the credibility of data protection. Culturally and linguistically adapted services were also documented as important equity considerations. Multilingual interfaces (eg, Chinese and Spanish) and culturally relevant scheduling (eg, adjustments for Lunar New Year) can accommodate diverse linguistic and cultural needs.

These solutions must be reinforced by broader policy frameworks and practical support systems to achieve scalable impact. The evidence supports subsidizing community technology training and retaining low-tech alternatives (such as telephone-based consultations and printed care instructions) to ensure access for those who cannot or do not wish to use digital channels. Hybrid care models that combine telemedicine with in-person options were endorsed across multiple studies as a pragmatic means of accommodating diverse needs without forcing a binary choice. Such interventions align with the WHO’s Decade of Healthy Aging (2021‐2030), which emphasizes digital inclusion as a pillar of equitable health care for older adults, aiming to protect their autonomy in digital health contexts.

### Future Directions

This scoping review mapped the dynamics of trust and vulnerability across individual, interpersonal, and systemic levels, highlighting targeted paths for future research. First, longitudinal studies are necessary to track changes in the dynamics of trust in telemedicine among older adults. Future research can investigate how trust in telemedicine evolves over time for older adults, particularly as they gain experience with digital tools.

Second, effectiveness can be better evaluated, such as training programs or age-friendly redesigns, and should be assessed in terms of their ability to reduce trust barriers and improve telemedicine adoption. Trust and vulnerability scales for telemedicine, based on empirical tests, have yet to be developed, indicating a need for more scientific measurement tools in future research.

Third, comparative cross-cultural and geographic comparisons remain insufficient. Future research should focus on understudied low- and middle-income countries and rural-urban divides to explore how systemic factors (eg, health care infrastructure) mediate trust-vulnerability links, thereby filling gaps in current regional data.

Fourth, future studies should examine how trust barriers differ across telemedicine modalities. The current evidence suggests that video consultations, wearable monitoring devices, telehealth apps, and electronic surveillance technologies may generate distinct trust concerns and vulnerability patterns. Comparative studies examining modality-specific trust dynamics would enable more targeted design and policy recommendations.

### Limitations

This review has certain limitations. The heterogeneous age thresholds used across included studies represent a substantive methodological limitation that affects the precision of the review’s conclusions. While justifications exist for this broader inclusion, the synthesis risks conflating the trust and vulnerability profiles of mid-life adults (50-64 y) with those of older adults (75+ y).

The search strategy constitutes a further methodological limitation. By requiring both “trust” and “vulnerability” as mandatory search terms, the review may have systematically excluded studies that address structurally similar phenomena under alternative conceptual labels. The evidence base synthesized here should, therefore, be understood as representing a theoretically bounded subset of the broader literature on older adults’ telemedicine engagement. Future reviews should consider expanding search terms to include “digital exclusion,” “digital divide,” “autonomy,” and “resistance” to capture this adjacent evidence.

In addition, most studies focused on primary medical care, overlooking particularly vulnerable populations, such as individuals with severe disabilities or those in underserved areas, thereby offering only a partial perspective on telemedicine experiences.

The restriction to English-language publications represents not only a practical constraint but also a potential source of cultural and regional bias. Studies conducted in non–English-speaking contexts may report trust barriers and vulnerability profiles that differ substantially from the predominantly Western and East Asian evidence synthesized here. Future reviews should deliberately include non-English literature to achieve more equitable geographic representation.

Another limitation concerns the aggregation of findings across heterogeneous telemedicine modalities. This review synthesized evidence from studies examining wearable devices, video consultations, telemedicine applications, electronic surveillance systems, and government digital health services under a unified thematic framework. While this approach enabled the identification of cross-cutting patterns, it necessarily obscures the trust dynamics specific to each modality.

Finally, the predominance of cross-sectional data restricted insight into the temporal evolution of trust and vulnerability.

### Conclusions

This review highlights that trust in telemedicine among older adults is shaped by a complex interplay of vulnerabilities across individual, interpersonal, and systemic levels. These dynamics show that the erosion of trust is not just a result of technological barriers but also involves bodily autonomy, social identity, and the capacity to adapt to systemic change.

To address these challenges, this study proposes an inclusive telemedicine improvement strategy. “Inclusion” in telemedicine does not always mean “high tech”; rather, meaningful inclusion requires interventions tailored to all 3 levels: intuitive tools and training to empower individuals, humanized interactions to strengthen interpersonal bonds, and systemic policies that balance digital innovation with low-tech alternatives and cultural responsiveness. By centering on such layered solutions, telemedicine can evolve into a more equitable tool that respects older adults’ diverse needs, improves health care equity, and enhances their healthcare experience by prioritizing trust, not just technology.

## Supplementary material

10.2196/84818 Multimedia Appendix 1 Study characteristics.

10.2196/84818 Checklist 1 PRISMA-ScR checklist.

## Abbreviations

PRISMA-ScR: Preferred Reporting Items for Systematic Reviews and Meta-Analyses Extension for Scoping Reviews
RQ: research question
WHO: World Health Organization
