# Supplementary material for: Trust Barriers and Vulnerabilities in Older Adults’ Telemedicine Adoption: Scoping Review
Source: Interact J Med Res. 2026 Jul 14;15:e84818. doi: 10.2196/84818 (PMC13367760; doi:10.2196/84818)
Supplement: Multimedia Appendix 1 [file ijmr-v15-e84818-s001.docx]

**Multimedia Appendix 1. Study characteristics.**

| No. | Authors & year | Journal | Title | Methods (study design) | Participants ’age | Sample size (included studies) | Geographic focus (Country) | Telemedicine modality | Trust barriers | Vulnerabilities | Trust barrier Codes | Vulnerability Codes |
| --- | --- | --- | --- | --- | --- | --- | --- | --- | --- | --- | --- | --- |
| 1 | Essén, 2008 | Social Science and Medicine | The two facets of electronic care surveillance: An exploration of the views of older people who live with monitoring devices | Qualitative research | ≥68 | 17 | Sweden | Electronic surveillance | Lack of understanding of monitoring technology's operation mechanism; Worry about monitoring data being viewed by others; Perception of remote monitoring as physical intrusion into personal space; Monitoring affecting personal decisions; Doubt about the necessity of monitoring services, considering existing manual checks sufficient | Limited knowledge of remote monitoring technology; Physical frailty and health risks; Obvious anxiety and low self-esteem; Dependence on care staff's services; Living alone and lacking immediate on-site support | T1, T2, T3, T4 | V1, V2, V3, V4 |
| 2 | Fitzsimmons et al., 2016 | BMC Health Services Research | Comparison of patient perceptions of telehealth-supported and specialist nursing interventions for early stage COPD: A qualitative study | Qualitative research | Mean=66.94 | 9 | the United Kingdom | Wearable devices | Initial concerns about telehealth technology use; Nervousness about telehealth technology operation; Concern about inability to use telehealth equipment correctly; Worry about telehealth data privacy and security; Doubt about telehealth equipment accuracy; Doubt about necessity of telehealth service (preferring traditional home nursing visits) | Limited knowledge of telehealth equipment; Family assistance in telehealth data entry; Physical frailty with progressive lung function decline and high exacerbation risk; Anxiety due to disease uncertainty; Dependence on clinicians for medication adjustment and health guidance; Difficulty in accessing timely care | T1, T2, T3, - | V1, V2, V3, V4 |
| 3 | Kruse et al., 2020 | JMIR Medical Informatics | Utilization barriers and medical outcomes commensurate with the use of telehealth among older adults - Systematic review | Systematic review | ≥50 | 57 | Europe, America | Video consultations; electronic surveillance; telemedicine apps | Low technical literacy, inability to navigate menus, lack of technology understanding; Lack of technical support; Visual acuity issues; Mental acuity issues; Hand-eye coordination problems; Auditory acuity issues; Computer anxiety; Lack of technology ownership; Privacy and security concerns; Lack of desire to use telehealth; Social implications concerns; Distrust of the internet | Limited telemedicine knowledge; Low self-management ability; Age-related physical declines; Chronic health conditions; Psychological distress; Dependence on external support; Fixed income and financial constraints; Inability to access timely healthcare | T1, T2, T3, T4 | V1, V2, V3, V4 |
| 4 | Ladin et al., 2021 | JAMA Network Open | Perceptions of telehealth vs in-person visits among older adults with advanced kidney disease, care partners, and clinicians | Qualitative research | ≥70 | 30 | the United States | No specified | Technical challenges; Hearing/vision impairments affecting telehealth engagement; Lack of quiet and private space for telehealth visits; Loss of interpersonal connection with clinicians; Difficulty in discussing bad news virtually; Racial/ethnic disparities in telehealth satisfaction; Concerns about telehealth care quality; Mistrust in telehealth; Low health literacy, inability to understand medical terms or manage health via telehealth; Access disparities; Language barriers | Limited telemedicine literacy; Complex health conditions, advanced CKD requiring frequent physical exams and lab tests; Age-related physical declines; Psychological vulnerability; Dependence on care partners; Socioeconomic limitations; Limited access to in-person backup care; Racial/ethnic vulnerabilities, lower trust in telehealth leading to delayed or inadequate care | T1, T2, -, T4 | V1, V2, V3, V4 |
| 5 | Schorr et al., 2021 | Circulation: Cardiovascular Quality and Outcomes | Harnessing mobile health technology for secondary cardiovascular disease prevention in older adults: A scientific statement from the American Heart Association | Systemic review | ≥65 | 26 | Europe, North America, Asia | Telemedicine apps; wearable devices; text messaging | Poor usability of mHealth tools (complex interfaces, difficult navigation); Low familiarity with mHealth technology (unfamiliar with smartphones/wearables); Age-related sensory/cognitive limitations (vision/hearing loss, slow information processing); Lack of technical support (no help with device setup, software updates); Privacy and security concerns (fear of health data leakage, cyberattacks); Social influence barriers (fear of technology replacing face-to-face interactions); Concerns about mHealth effectiveness (skepticism about behavior change and clinical outcomes); Preference for traditional care (prefer in-person visits with healthcare providers); Access disparities: High cost of mHealth devices/services (unaffordable for low-income older adults); Limited internet access (unstable connection in remote areas, no home internet) | Low mHealth literacy (unfamiliar with mHealth functions, unable to use tools for CVD management); Inadequate mHealth validation (consumer-grade devices may be inaccurate for CVD monitoring); Complex cardiovascular conditions (need frequent physical exams, lab tests); Age-related physical declines (motor function loss, shaky hands affecting device use); Psychological vulnerability (anxiety about virtual care, emotional distress from technology use) Psychological vulnerability (anxiety about technology); Dependence on external support (need family/caregivers for mHealth setup/operation) | T1, T2, T3, T4 | V1, V2, V3, V4 |
| 6 | Wilson et al., 2021 | BMC Public Health | Barriers and facilitators to the use of e-health by older adults: A scoping review | Scoping review | ≥60 | 14 | Europe, America, Australia | Telemedicine apps; online counseling | Low self-efficacy in e-health use (lack of confidence in operating e-health tools); Insufficient e-health knowledge and skills (no prior experience with e-health/technology); Poor e-health tool functionality (small screen/text/icons, complex navigation, faulty systems); Inadequate technological support (no training/troubleshooting help, unreliable family support); Privacy and security concerns (fear of health data leakage, worry about information access); Lack of social interaction (perceiving e-health as "inauthentic" without face-to-face contact); Cultural barriers (language issues, e-health conflicting with family time values); Mistrust in e-health (uncertainty about service providers); Preference for traditional healthcare (fear of traditional services disappearing, disbelief in e-health efficacy) | Low e-health literacy (unfamiliar with e-health functions, unable to interpret health data); Lack of e-health integration (no data sharing between e-health platforms and healthcare providers); Age-related physical impairments (reduced vision/hearing, poor fine motor control, shaky hands); Cognitive limitations (memory decline, slow information processing, difficulty learning new operations); Psychological vulnerability (anxiety about technology use, emotional distress from negative experiences); Dependence on external support (need family/caregivers for device setup/operation, reliance on coaches for training); Socioeconomic constraints (fixed pension income, inability to afford devices/internet upgrades)and geographical barriers (unreliable internet in rural/remote areas, limited access to in-person backup care) | T1, T2, T3, T4 | V1, V2, V3, - |
| 7 | Chen & Liu, 2022 | JMIR mHealth and uHealth | Assessing elderly user preference for telehealth solutions in China: Exploratory quantitative study | Quantitative research | ≥50 | 390 | China | Wearable devices | Low trust in telehealth data accuracy (doubt about data reliability; doctors refuse to use telehealth data for diagnosis/treatment adjustment); Preference for traditional in-person care (especially females and low-income groups; tendency to prioritize face-to-face doctor consultations) | Low telehealth literacy (unfamiliar with telehealth data application; unable to effectively use telehealth data for self-health management); Lack of system integration (telehealth data not integrated into hospital diagnosis process; unable to form continuous care); Chronic disease burden (need frequent health monitoring but lack reliable telehealth support); Dependence on external support (need children/grandchildren to assist with telehealth device operation and data interpretation; higher trust in telehealth when living with family); Socioeconomic limitations (fixed/low income; inability to bear telehealth costs not covered by basic medical insurance); Regional medical resource imbalance (high-quality medical/telehealth resources concentrated in tier-1/tier-2 cities; low-tier cities lack sufficient support) | -, T2, -, - | V1, V2, -, V4 |
| 8 | Hunter et al., 2022 | JMIR Formative Research | Enabling rural telehealth for older adults in underserved rural communities: Focus group study | Qualitative research | ≥55 | 98 | New Zealand | No specified | Low technology comfort level (difficulty operating devices, reliance on family for device setup/use); Security concerns (fear of online scams, worry about health data leakage); Privacy needs (desire to choose whether to have family present during telehealth consultations, especially for Māori); Cultural inappropriateness (Māori feeling uncomfortable accessing non-culturally safe health services); Preference for in-person relationships (willing to travel long distances to see regular GPs for consistent care, distrust in locums) | Low telehealth literacy (unfamiliar with telehealth concepts, unaware of telehealth benefits/devices); Lack of training/support (no regular community training on telehealth use, limited access to on-site technical support); Chronic disease management challenges (need frequent follow-ups, but telehealth fails to fully replace in-person monitoring due to connectivity/device issues); Dependence on family (rely on children/partners for device operation, missed opportunities to improve digital skills); Socioeconomic limitations (fixed/low income, unable to afford high-speed internet or advanced devices); Regional infrastructure gaps (poor rural digital infrastructure, slow progress of broadband initiatives); Limited service deployment (lack of fixed/mobile telehealth hubs, unable to access telehealth without community support) | T1, T2, T3, T4 | V1, V2, -, V4 |
| 9 | Kaihlanen et al., 2022 | BMC Health Services Research | Towards digital health equity - A qualitative study of the challenges experienced by vulnerable groups in using digital health services in the COVID-19 era | Qualitative research | ≥65 | 16 | Finland | Government service | Insufficient digital skills (inability to operate digital health platforms, poor usability of services); Lack of support/training (cancelled offline training during COVID-19; remote support is hard to access); Security concerns (fear of health data leakage, distrust in platform security); Privacy issues (no private space for remote consultations, e.g., family members overhearing); Preference for in-person care (perceive face-to-face services as more comprehensive for complex health issues; "old-school mind" favors physical consultations) | Inability to handle complex needs (digital services fail to address complex health issues, e.g., mental health therapy); Chronic disease management challenges (high users need frequent follow-ups but struggle with digital tools due to poor health); Dependence on others (rely on family for device operation, losing opportunities to improve skills); Economic vulnerability (unemployed and older adults cannot afford devices/internet) | T1, T2, T3, - | -, V2, -, V4 |
| 10 | Singh et al., 2022 | Technology in Society | Assessing the factors that influence the adoption of healthcare wearables by the older population using an extended PMT model | Quantitative research | ≥60 | 534 | India | Wearable devices | Low digital skills (struggle with smartwatch operation and app functions); Lack of support and training (few resources for learning to use HWDs); Fear of new technology (hesitate to try HWDs due to fear of failure); Lack of confidence (doubt their ability to use HWDs correctly); Data privacy concerns (fear of health data leakage, especially in IoT devices); Security issues (doubts about the security of device connections) ; Social influence (opinions from peers and family affect attitude towards HWDs) | Poor understanding of HWDs (unaware of device functions and benefits); Difficulty in evaluating information (unable to judge the accuracy and reliability of health data); Chronic disease burden (need continuous health monitoring but face challenges with HWDs); Multiple comorbidities (complicate the use of HWDs and health management); Economic dependence (rely on family for financial support to buy HWDs); Technical dependence (depend on others for device operation and problem - solving) | T1, -, T3, - | V1, V2, V3, V4 |
| 11 | Korkmaz Yaylagul et al., 2022 | International Journal of Environmental Research and Public Health | Trends in telecare use among community-dwelling older adults: A scoping review | Scoping review | ≥65 | 40 | Europe, North America, Asia | Video consultations; electronic surveillance; telemedicine apps; wearable devices | Low technology acceptance (complex telecare devices, difficulty in operation and understanding); Poor device usability (unsuitable design for older adults, e.g., unclear interfaces); Lack of technical support (insufficient training for users and caregivers on device use); Privacy intrusion (surveillance devices like cameras are perceived as threatening personal privacy); Lack of data security trust (doubts about the security of health data collected by telecare); Stigma of telecare use (perceiving telecare as a sign of dependence, leading to social embarrassment); Reduced face-to-face interaction (fear of loneliness due to less in-person care); Preference for in-person care (distrust in telecare’s ability to replace face-to-face diagnosis and care) | Unawareness of telecare benefits (lack of knowledge about telecare’s role in chronic disease management); Inability to evaluate telecare services (cannot judge whether telecare meets personal health needs); Chronic disease management challenges (need continuous monitoring but telecare fails to fully address advanced-stage conditions, e.g., severe dementia); Frailty-related limitations (physical/cognitive frailty hinders independent use of telecare devices); Fear of dependence (seeing telecare as reducing independence, leading to resistance); Cognitive barriers (memory decline affects remembering device operation steps); Dependence on caregivers (rely on family/caregivers for device setup and problem-solving); Economic vulnerability (fixed/low income limits access to paid telecare services); Institutional support gaps (local governments lack policies to subsidize telecare for disadvantaged older adults); Regional resource imbalance (scarce telecare resources in underserved areas) | T1, T2, T3, T4 | V1, V2, V3, V4 |
| 12 | Guo et al., 2023 | Digital Health | The influence of health information attention and app usage frequency of older adults on persuasive strategies in mHealth education apps | Quantitative research | ≥50 | 111 | China | Telemedicine apps | Fear of new technology; Privacy concerns; Complex app functions (overwhelming information, difficulty in navigating MHE apps); Low receptivity to social role strategies (distrust in app-assumed social roles); Preference for traditional health education (reliance on offline health guidance, low recognition of MHE app effectiveness) | Poor MHE app literacy (unable to effectively use app functions for health education); Inability to evaluate persuasive strategies (cannot judge which app strategies fit personal health needs); Cognitive decline (memory loss affecting remembering app operation steps); Low motivation for sustained use (easy to lose interest in MHE apps, difficulty forming usage habits); Dependence on external guidance (need family/caregivers to assist with app setup and use); Susceptibility to social influence (reliance on peer/social group feedback to use MHE apps) | T1, T2, T3, - | V1, V2, V3, - |
| 13 | Liu et al., 2023 | BMC Geriatrics | The role of trust and habit in the adoption of mHealth by older adults in Hong Kong: A healthcare technology service acceptance (HTSA) model | Quantitative research | ≥65 | 201 | China | Telemedicine apps | Fear of technology misuse (hesitation to try mHealth due to worry about incorrect operation); Lack of trust in data security (doubts about the security of sensitive health data in mHealth apps); Preference for traditional healthcare (reliance on offline medical services, low recognition of mHealth effectiveness) | Poor mHealth literacy (inability to effectively understand and use mHealth functions for health management); Inability to evaluate mHealth value (difficulty in judging whether mHealth meets personal health needs); Low motivation for initial adoption (lack of drive to start using mHealth, easy to give up due to minor difficulties); Habit formation difficulty (hard to develop regular mHealth use habits without external incentives); Dependence on external trust-building (rely on service quality, government policies, and social influence to establish trust in mHealth) | T1, T2, T3, - | V1, V2, V3, - |
| 14 | Orzechowski et al., 2023 | Sensors | User perspectives of geriatric German patients on smart sensor technology in healthcare | Qualitative research | ≥73 | 11 | Germany | Wearable devices | Resistance to technology adoption; Third-party data access concerns (fear that health insurers may access sensor-collected data); Fear of reduced personal contact (worries that sensors may decrease face-to-face communication with healthcare professionals); Concern about reduced decision involvement (anxiety about being excluded from therapeutic decision-making); Preference for healthcare professionals' subjective judgment (some patients trust doctors' experience more than sensor data) | Poor understanding of sensor data; Cost burden concerns; Dependence on healthcare professionals' guidance; Dependence on financial support | -, T2, T3, T4 | V1, V2, -, - |
| 15 | Ali et al., 2024 | International Journal for Equity in Health | Strategies to optimise the health equity impact of digital pain self‑reporting tools: A series of multi‑stakeholder focus groups | Qualitative research | ≥65 | 6 | the United Kingdom | Telemedicine apps | Digital access barriers; Low digital literacy (difficulty operating tools, e.g., older adults struggling with app navigation); Poor tool usability (unclear instructions, lack of accessibility features like zoom for visual impairments); Data privacy anxiety (fear of unauthorized access to pain data, e.g., ethnic minorities worried about third-party data use); Confidentiality concerns (fear of data leaks affecting personal life); Discomfort in virtual settings; Preference for face-to-face pain reporting | Language barriers (no multilingual support, e.g., ethnic minorities with limited English struggling with English-only tools); Lack of supportive resources (no guidance for tool use, e.g., people with low literacy unable to understand instructions); Poor pain terminology understanding (unable to distinguish pain types like "sharp" vs "dull", e.g., ethnic minorities unfamiliar with medical terms); Inability to interpret tool outputs (unable to understand pain reports, e.g., older adults confused by data summaries); Dependence on others for tool operation (needing family/peers to help with devices, e.g., older adults relying on children to use apps); Dependence on external resources (needing community support for device access, e.g., low-income groups relying on public libraries for internet) | T1, T2, T3, T4 | V1, V2, -, - |
| 16 | Chen et al., 2024 | Healthcare | Understanding the role of technology anxiety in the adoption of digital health technologies (DHTs) by older adults with chronic diseases in Shanghai: An extension of the unified theory of acceptance and use of technology (UTAUT) model | Quantitative research | ≥60 | 309 | China | No specified | High technology anxiety (unease, confusion, or nervousness when using DHTs); Negative smartphone experience (bad past experiences with smartphones reduce trust in DHTs relying on smartphones); Lack of DHTs experience (anxiety towards unfamiliar DHTs due to no prior use); Concern about data security (worries about data breaches and privacy leaks in DHTs); Negative social influence (digital divide makes older adults feel incompetent in DHTs, reducing adoption intention) | Low education level (lower digital literacy due to low education, hindering DHTs use); Poor DHTs operation ability (difficulty learning and using DHTs due to low digital literacy); Susceptibility to negative social influence (easily affected by digital divide-related incompetence, reducing use willingness); Dependence on family assistance (rely on younger family members for DHTs operation); Dependence on external technical support (need community/medical institution training for DHTs use); Dependence on facilitating conditions (rely on sufficient software, network, and hardware resources to use DHTs) | T1, -, T3, T4 | V1, V2, V3, V4 |
| 17 | Chu et al., 2024 | Journal of Renal Care | Exploring the factors affecting home dialysis patients' participation in telehealth-assisted home visits: A mixed-methods study | Quantitative & qualitative research | Survey-68±11; Interview-65±16 | 55 | Australia | Family therapy | Lack of telehealth training/instructions (insufficient knowledge on how to use telehealth, e.g., no guidance on virtual assessments); Poor technological usability; Inability to conduct virtual physical assessments (unconfident in self-performing assessments like fluid checks, doubting accuracy); Reduced social interaction; Privacy concerns; Entrenched traditional care beliefs; Dependence on clinician guidance | Language barriers; Poor telehealth operation skills; Disability-related barriers; Geographic limitations (rural patients facing poor internet access, e.g., no NBN connection); Dependence on nurse social interaction (relying on home visits for social contact, especially for lonely patients); Dependence on technical support (needing others to assist with device troubleshooting) | T1, T2, T3, T4 | V1, V2, -, V4 |
| 18 | Fang et al., 2024 | Humanities and Social Sciences Communications | Empowering older adults: Bridging the digital divide in online health information seeking | Quantitative research | ≥60 | 277 | China | Virtual diagnostics; telemedicine apps; wearable devices | Low digital skills (lack of basic skills like information acquisition, struggling with smartphone operation); Lack of past experience (no successful experience in online health information seeking, leading to fear of failure); Insufficient self-efficacy (doubt in own ability to seek online health information); Negative emotional state (anxiety, resistance towards digital technology, reducing willingness to seek information); Privacy | Poor online health information evaluation ability; Inability to apply online information (failing to translate acquired online health knowledge into practical health behaviors); Lack of digital health knowledge; Distrust in online information (doubting the accuracy and credibility of online health content); Resistance to digital behavior change (unwilling to shift from traditional to online health information seeking); Dependence on direct experience; Dependence on emotional support; Dependence on peer/model learning; Dependence on effective verbal persuasion | T1, -, T3, T4 | V1, V2, V3, - |
| 19 | Gallardo et al., 2024 | Gerontology and Geriatric Medicine | The role of initial trust in the behavioral intention to use telemedicine among Filipino older adults | Quantitative research | ≥60 | 180 | the Philippines | Telemedicine apps | Low perceived behavioral control (lack of self-assessed knowledge and ability to use telemedicine independently); Limited telemedicine experience (most used telemedicine only 1-2 times, leading to unfamiliarity with operations); Technical issues concerns (worries about device compatibility, network instability, etc., affecting trust in telemedicine functionality); Concern about medical data confidentiality (fear that telemedicine fails to protect sensitive health information, reducing initial trust)  Dependence on offline care habits (preference for in-person consultations due to long-term trust in traditional healthcare) | Low educational level (45% with high school or below education, limiting understanding of telemedicine benefits); Poor telemedicine operation skills (unable to independently complete virtual consultations, need family assistance); Inability to evaluate telemedicine reliability (unable to judge whether telemedicine meets medical needs, e.g., doubts about remote diagnosis accuracy); Lack of un derstanding of telemedicine benefit (insufficient awareness of telemedicine’s convenience, cost-saving advantages)  Mobility limitations in older groups over 76 (higher need for telemedicine due to transportation difficulties, but limited by technology access)  Low initial trust sensitivity (easily affected by negative information about telemedicine, e.g., data leakage news); Dependence on social approval (rely on family/friends’ opinions to decide on telemedicine use, lack of independent judgment); Resistance to behavior change (unwilling to shift from traditional to telemedicine care models)  Dependence on family technical assistance (need family members to set up devices, operate platforms for telemedicine); Dependence on expert guidance (rely on doctors/health workers’ recommendations to build trust in telemedicine); Dependence on positive social norms (need consistent approval from family/friends to enhance telemedicine adoption intention) | T1, T2, T3, - | V1, V2, V3, V4 |
| 20 | Li et al., 2024 | BMC Geriatrics | Effect of older adults willingness on telemedicine usage: An integrated approach based on technology acceptance and decomposed theory of planned behavior model | Quantitative research | ≥60 | 400 | China | No specified | Low perceived ease of use (perceiving telemedicine operation as difficult, e.g., struggling with app navigation or video consultation setup); Insufficient self-efficacy (doubting own ability to independently use telemedicine, e.g., unable to troubleshoot technical issues); Technical risk concerns (worries about telemedicine functional failure, e.g., poor video quality affecting diagnosis); Fear of personal health information leakage or improper use, reducing trust in telemedicine; Lack of face-to-face interaction (anxiety from no in-person communication with medical staff); Preference for offline medical services (trusting in-person diagnosis and physical examinations more than remote consultations) | Poor telemedicine function understanding (unable to use core features, e.g., booking appointments or uploading health reports); High risk sensitivity (easily affected by emotional/cost risks, e.g., anxiety from potential data leakage); Dependence on technical assistance (need family/friends to help with telemedicine setup and operation); Dependence on social approval (need consistent support from family/community to adopt telemedicine) | T1, T2, T3, T4 | V1, V2, V3, - |
| 21 | Niu et al., 2024 | Healthcare | How expectations and trust in telemedicine contribute to older adults’ sense of control: An empirical study | Quantitative research | ≥60 | 661 | China | Government service | Low digital literacy (limited understanding of telemedicine technology, e.g., unable to operate multi-device collaboration like smartphones + electronic monitors); Resistance to new technology (inherent hesitation to adopt telemedicine due to unfamiliarity with digital products); Safety trust deficiency (fear of personal health information leakage or improper use, e.g., worrying about medical records being accessed by unauthorized parties); Lack of face-to-face interaction (anxiety from no in-person communication with medical staff, reducing trust in telemedicine effectiveness); Preference for offline medical services (trusting in-person diagnosis and physical examinations more than remote consultations, e.g., believing face-to-face visits ensure more accurate symptom judgment) | Poor multi-function use ability; Inability to transfer skills (; High safety sensitivity (easily influenced by privacy/security risks); Low sense of digital control (feeling powerless in digital life, reducing adoption motivation); Dependence on other’s support | T1, T2, T3, T4 | V1, V2, V3, - |
| 22 | Turcotte et al., 2024 | Australasian Journal on Ageing | Factors influencing older adults' participation in telehealth interventions for primary prevention and health promotion: A rapid review | Rapid review | ≥50 | 24 | Europe, North America, Asia | Telemedicine apps; video consultations; wearable devices | Privacy anxiety (feeling telehealth is invasive, e.g., worrying about unauthorized access to health data collected via wearables); Lack of social dimension (feeling isolated due to no in-person interaction with peers or providers, e.g., missing group support in online exercise programs); Fear of replacing human contact (worrying telehealth will substitute in-person care, reducing trust in provider-patient relationships); Unfamiliar providers (distrust in unknown healthcare professionals delivering telehealth services); Preference for offline preventive/promotive care | Poor technology adaptation; Age-related impairments; Lack of self-efficacy; Hedonic motivation deficiency; Habit resistance (unwilling to integrate telehealth into daily routines, e.g., refusing to replace traditional exercise with online sessions); Dependence on technical assistance; Dependence on social interaction; Dependence on familiar providers (relying on trusted in-person doctors to recommend telehealth, otherwise refusing to try) | T1, T2, T3, T4 | V1, V2, V3, V4 |
| 23 | Windle et al., 2024 | JBI Evidence Implementation | Factors that influence the implementation of innovation in aged care: A scoping review | Scoping review | Most≥65 | 193 | Europe, North America, Australia | No specified | Low capabilities (lack of skills/knowledge for innovation); Low adaptability (rigid innovation not fitting aged care context, e.g., generic exercise programs not adjusted for residents with mobility impairments); Complex remote monitoring | Digital health tools not considering low vision or dexterity issues; Consumer voice absence (older adults’ preferences ignored, e.g., digital health tools not considering low vision or dexterity issues) | T1, T2, -, - | V1, V2, -, V4 |
| 24 | Husain & Greenhalgh, 2025 | Journal of Internet Medical Research | Examining intersectionality and barriers to the uptake of video consultations among older adults from disadvantaged backgrounds with limited English proficiency: Qualitative narrative interview study | Qualitative research | ≥65 | 17 | the United Kingdom | Video consultations | Complex digital pathways (confusion about multiple access points, e.g., not knowing whether to use NHS app, website, or phone call for appointments); Fear of disorientation (anxiety from navigating unfamiliar digital platforms, leading to avoidance of video consultations); Weakened therapeutic relationship (lack of in-person rapport, e.g., feeling doctors are "distracted" or "disengaged" through screens); Absent presence (diminished sense of connection due to delayed responses, mechanical intonation, and limited nonverbal cues) | Limited English proficiency; Inability to navigate digital systems; Lack of technical troubleshooting skills (unable to resolve minor issues like poor network, leading to consultation abandonment); Physical impairments; Cognitive challenges (memory decline, struggling to remember multiple steps for video consultations); Thinking doctors don’t "care" without in-person interaction; Reduced agency (feeling powerless to advocate for oneself, e.g., not daring to question doctors due to language/digital barriers); Socioeconomic constraints; Geographical/institutional gaps (no targeted support for disadvantaged groups); Dependence on family assistance t; Dependence on trusted providers | T1, -, -, T4 | V1, V2, -, V4 |
| 25 | Tan et al., 2025 | JMIR Aging | Determinants of telehealth adoption among older adults: Cross-sectional survey study | Quantitative research | ≥60 | 119 | Malaysia | Video consultations; Wearable devices; government service | Low perceived ease of use (finding telehealth hard to operate, e.g., unable to navigate user interfaces or complete online appointment steps); Negative attitude toward technology (holding unfavorable views on telehealth) ; Inertia (resistance to changing traditional healthcare habits, e.g., preferring in-person visits over learning to use telehealth); Low subjective well-being perception (doubting telehealth improves quality of life, e.g., feeling telehealth lacks the "human touch" of in-person care); Cultural preference for in-person care (valuing face-to-face communication due to cultural norms, e.g., believing in-person consultations build stronger doctor-patient trust)  Strong preference for offline healthcare (trusting in-person diagnosis and physical examinations more, e.g., believing doctors can better assess symptoms face-to-face) | Poor digital literacy (lack of skills to use telehealth tools, e.g., unable to troubleshoot minor technical issues like poor network connections); Lack of technical problem-solving skills (unable to fix simple issues like app crashes, leading to abandoned telehealth sessions); High resistance to change (unwilling to adapt to new healthcare modes, e.g., refusing to try telehealth even if it saves travel time)  Dependence on family assistance (needing relatives for telehealth operation, e.g., children helping with appointment booking or symptom description); Dependence on institutional support (relying on healthcare providers for guidance, e.g., needing doctors to explain how to use telehealth tools); Dependence on external resources (needing affordable technology, e.g., relying on government subsidies to buy smartphones for telehealth) | T1, T2, -, T4 | V1, V2, V3, V4 |
| 26 | Zhu et al., 2025 | Digital Health | How eHealth use and cancer information-seeking influence older adults’ acceptance of genetic testing: Mediating roles of PIGI and cancer worry | Quantitative research | ≥60 | 1852 | the United States | No specified | Poor digital operation ability (struggling to navigate genetic testing-related platforms, e.g., unable to complete online genetic test appointment or result inquiry); Preference for traditional health screening (trusting conventional physical examinations more than genetic testing, e.g., believing blood tests or imaging are more accurate for disease detection) | Low eHealth literacy (inability to effectively use eHealth tools for genetic information access, e.g., unable to search for genetic testing details or communicate with providers online); Emotional vulnerability to cancer information (easily influenced by negative cancer information, leading to distrust in genetic testing); Dependence on others for eHealth operation (needing family or providers to assist with eHealth tools, e.g., children helping with online genetic test consultation); Dependence on professional guidance (relying on doctors to recommend genetic testing, otherwise refusing to try, e.g., only considering genetic testing if advised by a trusted physician); Dependence on social approval (needing others’ recognition to accept genetic testing, e.g., only undergoing genetic testing if family members support it) | T1, T2, -, - | V1, V2, V3, - |
| 27 | Adams et al., 2025 | Journal of Internet Medical Research | Digital interventions for older people experiencing homelessness: Systematic scoping review | Scoping review | ≥50 | 10 | America | Telemedicine apps; government service | Poor technical experience (unreliable internet, confusing interfaces, e.g., internet disconnects during telecare visits or unable to navigate vocational training apps); Impersonal digital interactions (feeling detached from care providers via digital platforms, e.g., perceiving telehealth as "lacking human touch" compared to in-person visits); Social isolation reinforcement (digital interventions reducing in-person social contact, e.g., preferring video visits over in-person interactions leading to less face-to-face support); Preference for in-person care (trusting face-to-face services more, e.g., believing in-person consultations with doctors provide more comprehensive health assessments than telecare) | Low digital literacy (inability to operate digital devices/tools, e.g., struggling to use tablets for video consultations or text message reminders); Poor understanding of intervention goals (failing to grasp how digital tools support health/social needs, e.g., not knowing vocational training apps help with job searches); Lack of troubleshooting skills (unable to resolve minor issues, e.g., not knowing how to reconnect to Wi-Fi after disconnection during a video call); Resistance to change (unwilling to switch from traditional to digital services, e.g., refusing to use telecare despite mobility difficulties); Low self-efficacy (no confidence in using digital tools, e.g., hesitating to try smoking cessation apps due to fear of "doing it wrong"); Emotional vulnerability to technical failures (feeling frustrated/anxious when digital tools malfunction, e.g., abandoning telecare after repeated internet disruptions); Dependence on organizational support (needing staff assistance for digital interventions, e.g., shelter workers preconfiguring tablets with videoconferencing software); Dependence on peer support (relying on peers to troubleshoot/learn digital tools, e.g., other homeless adults helping with text message setup); Dependence on external resources (needing donated devices/internet access, e.g., relying on charities to provide tablets or free Wi-Fi in shelters) | T1, T2, -, T4 | V1, V2, V3, - |
| 28 | Fothergill et al., 2025 | BMC Geriatrics | Understanding how, for whom and under what circumstances telecare can support independence in community - dwelling older adults: A realist review | Realist review | Unreported | 32 | Europe, America, Australia | Pendant alarms; monitoring sensors; wearable devices; electronic surveillance | Low technical understanding (unclear how telecare works or responds to emergencies, e.g., not knowing who will assist when an alarm is triggered)（; Poor device adaptability (inability to use unfamiliar telecare designs, e.g., struggling with non-tablet-style devices if only familiar with tablets); Unreliable functionality (false alarms or unresponsive devices, e.g., fall detectors being too sensitive/insensitive leading to distrust); Resistance to invasive monitoring (reluctance to accept passive/ambient sensors, e.g., viewing 24-hour behavioral monitoring as an intrusion of personal space); Anxiety about data usage (worries about who accesses telecare data, e.g., fearing health data collected by sensors is shared without consent); Stigma of frailty (perceiving telecare use as a sign of being "frail" or "vulnerable," e.g., avoiding pendant alarms to avoid being seen as dependent); Reduced in-person interaction (fear telecare replaces face-to-face support, e.g., worrying video consultations reduce opportunities for in-person emotional connection); Low perceived utility (doubting telecare’s benefits for independence, e.g., not believing health deterioration detection helps maintain daily living); Preference for in-person care (trusting face-to-face health assessments more, e.g., believing in-person doctor visits provide more accurate health evaluations than telecare monitoring) | Poor tech adaptability (struggling with complex interfaces, e.g., unable to navigate multi-functional health platforms); Inclusive design lack (tech not adapting to disabilities, e.g., visually impaired users unable to access text-only health websites); Distrust in data usage (worries about sensitive info being shared, e.g., fearing genetic data from testing apps is misused by third parties); Anxiety about algorithm bias (doubting fair data analysis, e.g., low-income groups fearing algorithm-driven apps prioritize affluent users); Unclear data policies (confusion about data collection scope, e.g., not knowing what health behaviors are tracked by fitness apps); Stigma of tech reliance (perceiving digital health use as "needing help," e.g., older adults avoiding health apps to avoid being seen as "incompetent"); Reduced in-person trust (fear digital replaces human care, e.g., patients doubting video consultations are as effective as in-person visits); Low perceived benefit (doubting digital health improves outcomes, e.g., low-income groups not believing diet apps help with long-term eating changes); Preference for in-person care (trusting face-to-face diagnosis more, e.g., believing doctors’ in-person assessments are more accurate than AI-driven app diagnoses) | T1, T2, T3, T4 | V1, -, V3, V4 |
| 29 | Western et al., 2025 | Health Psychology and Behavioral Medicine | Bridging the digital health divide: A narrative review of the causes, implications, and solutions for digital health inequalities | Narrative review | Unreported | Unreported | Global | Social media service; telemedicine apps; wearable devices | Poor tech adaptability (struggling with complex interfaces, e.g., unable to navigate multi-functional health platforms); Inclusive design lack (tech not adapting to disabilities, e.g., visually impaired users unable to access text-only health websites); Distrust in data usage (worries about sensitive info being shared, e.g., fearing genetic data from testing apps is misused by third parties); Anxiety about algorithm bias (doubting fair data analysis, e.g., low-income groups fearing algorithm-driven apps prioritize affluent users); Unclear data policies (confusion about data collection scope, e.g., not knowing what health behaviors are tracked by fitness apps); Stigma of tech reliance (perceiving digital health use as "needing help," e.g., older adults avoiding health apps to avoid being seen as "incompetent"); Reduced in-person trust (fear digital replaces human care, e.g., patients doubting video consultations are as effective as in-person visits); Low perceived benefit (doubting digital health improves outcomes, e.g., low-income groups not believing diet apps help with long-term eating changes); Preference for in-person care (trusting face-to-face diagnosis more, e.g., believing doctors’ in-person assessments are more accurate than AI-driven app diagnoses) | Low digital health literacy (inability to find/interpret digital health info, e.g., struggling to understand medical data on health apps); Outdated digital skills (unfamiliar with new tech like social media or wearables, e.g., not knowing how to use smartwatch health monitoring); Poor health info appraisal (unable to judge info credibility, e.g., believing misleading health claims on unregulated apps); Cognitive impairment impacts (memory loss affecting tech use, e.g., dementia patients forgetting how to log health symptoms on apps); Physical limitation barriers (mobility/sensory issues limiting interaction, e.g., arthritis patients unable to tap small app buttons); Chronic disease management gaps (digital tools not adapting to multiple conditions, e.g., diabetes patients unable to integrate blood sugar data with heart monitoring apps); Resistance to digital change (unwilling to switch from traditional care, e.g., middle-aged adults refusing telemedicine despite time-saving benefits); Low self-efficacy in tech (no confidence in digital health use, e.g., less educated groups hesitating to try online symptom checkers); Emotional vulnerability to negative experiences (abandoning tech after failures, e.g., users quitting health apps after repeated login errors); Dependence on family assistance (needing relatives to explain tech functions, e.g., children helping parents understand prescription refill apps); Dependence on professional guidance (relying on doctors for tech setup, e.g., nurses configuring remote monitoring devices for patients); Dependence on community resources (needing public facilities for access, e.g., homeless populations relying on library Wi-Fi to use health portals) | T1, T2, T3, T4 | V1, V2, V3, V4 |
| 30 | Zhang et al., 2025 | Journal of Medical Internet Research | Social media and eHealth literacy among older adults: Systematic literature review | Systematic review | ≥50 | 16 | Asia, North America | Social media service; telemedicine apps | Limited platform adaptability (preferring single/familiar platforms); Poor tech troubleshooting (unable to resolve minor issues, e.g., not knowing how to fix login failures for social media health accounts); Financial security anxiety (fear of online payment risks, e.g., worrying about money loss when paying for health services via social media); Data misuse worry (anxiety about sensitive info sharing, e.g., fearing health records shared on social media are used by third parties without consent); Stigma of tech reliance (perceiving social media use for health as "needing help," e.g., avoiding online health consultations to avoid being seen as "incompetent"); Low trust in health info (doubting social media info credibility, e.g., not believing COVID-19 prevention tips from non-authoritative accounts); Reduced in-person interaction fear (worrying social media replaces face-to-face support, e.g., hesitant to use social media for health communication due to preference for in-person doctor visits); Preference for in-person health services (trusting offline care more, e.g., believing in-person physical examinations are more accurate than health advice from social media) | Low digital skills (inability to operate social media functions, e.g., struggling to share health information or join online health groups); Lack of interactive health skills (unable to communicate health needs online, e.g., not knowing how to describe symptoms clearly in social media health groups); Poor health info appraisal (unable to judge info reliability, e.g., unable to distinguish false from true health rumors on social media); Cognitive impairment impacts (memory loss affecting social media use, e.g., forgetting how to record daily health data on social media health apps); Physical limitation barriers (mobility/sensory issues limiting interaction, e.g., arthritis patients unable to tap small buttons on social media health pages); Resistance to digital change (unwilling to switch to social media health tools, e.g., refusing to use social media for health management despite convenience); Low self-efficacy in eHealth (no confidence in social media health use, e.g., hesitating to try online health interventions due to fear of "doing it wrong"); Emotional vulnerability to misinformation (abandoning healthy behaviors after false info, e.g., quitting medication after seeing misleading "natural cure" posts on social media); Dependence on family assistance (needing relatives to guide social media use, e.g., children helping parents understand health info on social media); Dependence on peer support (relying on peers for health info verification, e.g., asking other older adults in social groups to confirm health advice); Dependence on institutional guidance (needing authoritative organizations for info, e.g., only trusting health info from hospital official social media accounts) | T1, T2, T3, T4 | V1, V2, V3, - |
